# Supplementary material for: FLS2–RBOHD–PIF4 Module Regulates Plant Response to Drought and Salt Stress
Source: Int J Mol Sci. 2022 Jan 19;23(3):1080. doi: 10.3390/ijms23031080 (PMC8835674; doi:10.3390/ijms23031080)
Supplement: Supplementary file 1 [file ijms-23-01080-s001.zip › Supplemental data FLS2.pdf]

Supplemental Tables

Table S3. List of mutant lines.

Gene name	accession number	Mutant lines
PIF4	AT2G43010	SAIL_1288_E07
RBOHD/RBOHF	AT5G47910/AT1G64060	CS9558
		CS68522
FLS2	AT5G46330	SALK_099606, SALK_141277

Table S4. List of oligonucleotides.

**Gene**	**Name**	**Sequence (5' ------> 3')**
**Mutant confirmed**		
FLS2	Fls2-9906LP	TCAAGGAAACAAATTCAACGG
	Fls2-9906RP	AACGATGGGAAAACCATATCC
	Fls2-1227LP	AGGGCTTCTTACAAACCTTCG
	Fls2-1227RP	CGTTGATGTTTTTGAACACCC
PIF4	Pif4LP	AATACATTTTGCAGGCAATCG
	Pif4RP	CGTAATGAAGTTGCACGTTTACTC
	SalkLB	ATTTTGCCGATTTCGGAAC
	SailLB	TAGCATCTGAATTTCATAACCAATCTCGATAC
**Semi-quantitative RT-PCR**		
PIF4	PIF4rts	ATTATGGCGAGATGGACAAG
	PIF4rta	AAGTGGGAGGAGAAGTCGTC
actin	actins	AACTGGGATGATATGGAGAA
	actina	CCTCCAATCCAGACACTGTA

Supplemental Figures

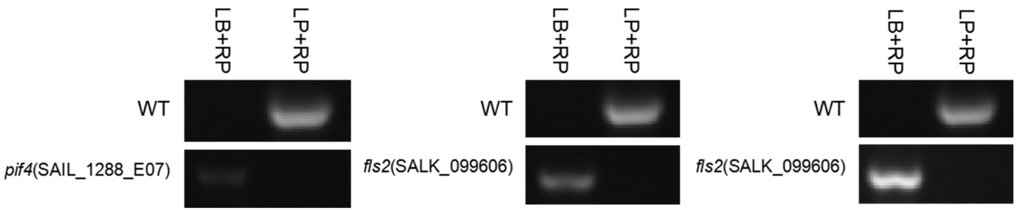

Figure S1. Verification of the T-DNA insertion of mutants. DNA was extracted from the mutants and WT; the location of the T-DNA insertion in each mutant was verified by PCR with the corresponding primers. (A) Identification of the homozygotes of mutants of *pif4*, *fls2-1*, and *fls2-2*.

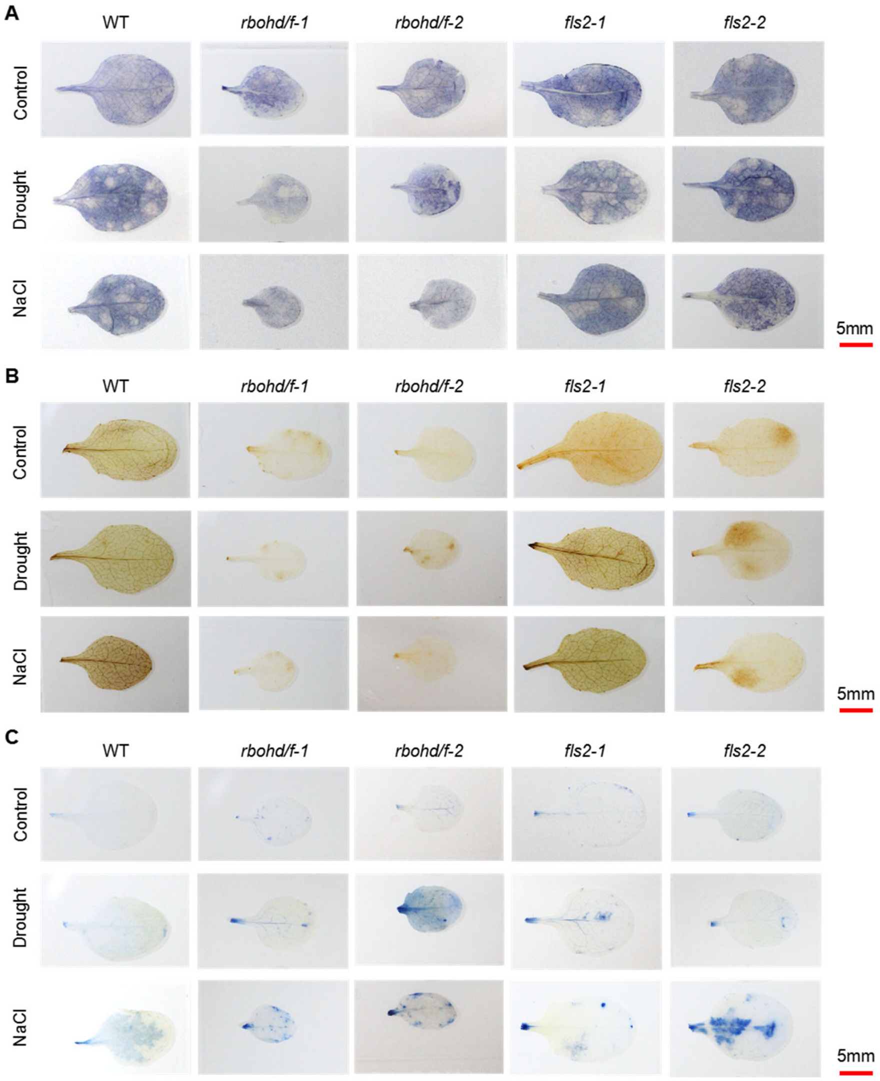

Figure S2. Analysis of the accumulation of ROS and apoptosis under drought and salt stress conditions. (A) The accumulation of O^{2-} in leaves of WT, *fls2* mutant, and *rbohd rbohf* double

mutant (rbohd_f) was detected by NBT staining, untreated samples were used as control. (B) The content of H_2O_2 in leaves of WT, *fls2* mutant, and *rbohd rbohf* double mutant (rbohd_f) was detected by DAB staining, untreated samples were used as control. (C) Trypan blue was used to detect apoptosis in leaves of WT, *fls2* mutant, and *rbohd rbohf* double mutant (rbohd_f), untreated samples were used as control. The scale bar is 5 mm.

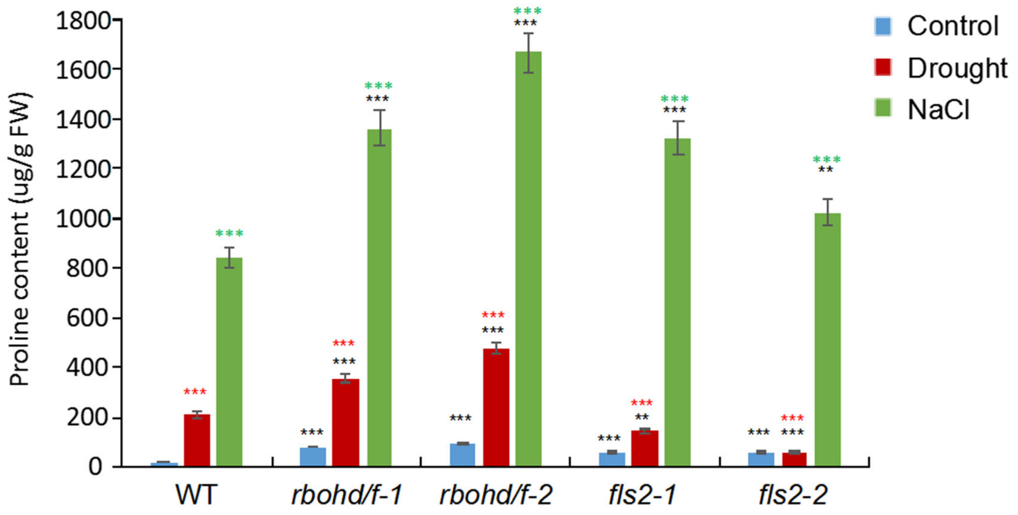

Figure S3. Analysis of the accumulation of proline under drought and salt stress conditions. The proline content in leaves of WT, *fls2* mutant, and *rbohd rbohf* double mutant (rbohd_f) treated with drought and salt stress for one week was determined, untreated samples were used as control. The data were analyzed by one-way ANOVA following Brown-Forsythe test. **: $p < 0.01$, ***: $p < 0.001$. The black stars represent the comparison between mutant and WT; red stars represent the comparison between drought and control; green stars represent the comparison between NaCl and control.

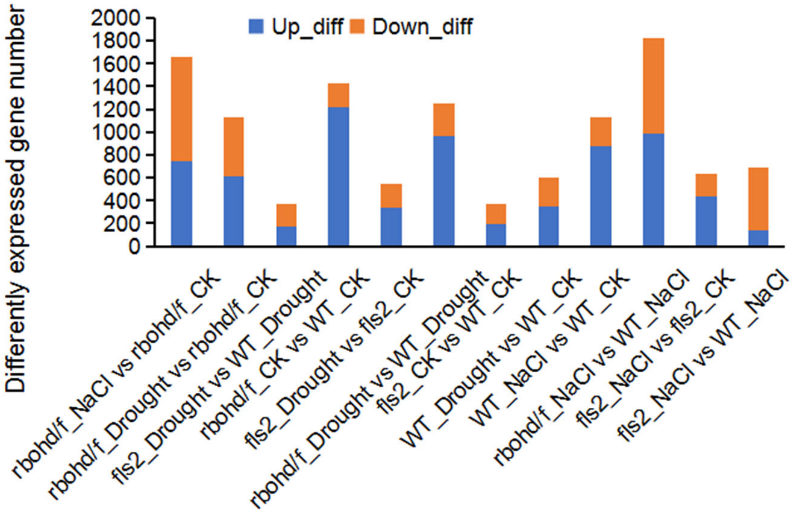

Figure S4. Identification of differentially expressed genes in different comparison groups. Statistical analysis of the number of differentially expressed genes in different comparison groups. The red histogram indicates up-regulated genes, and the blue histogram indicates down-regulated genes. CK: control.

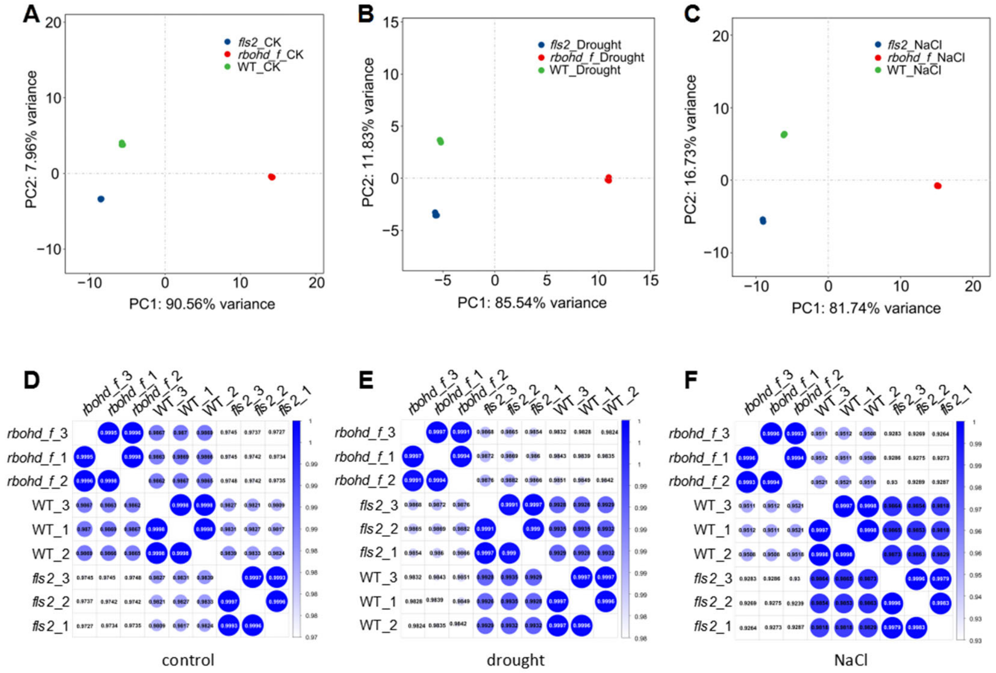

Figure S5. The principal component analysis and correlation coefficient analysis among different samples. (A-C) The principal component analysis under normal, drought, and salt treatment conditions, with 3 biological repetitions of each sample. (D-F) The correlation coefficient analysis under normal, drought, and salt treatment conditions. The three biological repetitions of each sample are symmetrically distributed in the fixed areas on both sides of the separation line. The darker the blue, the higher the correlation between samples.

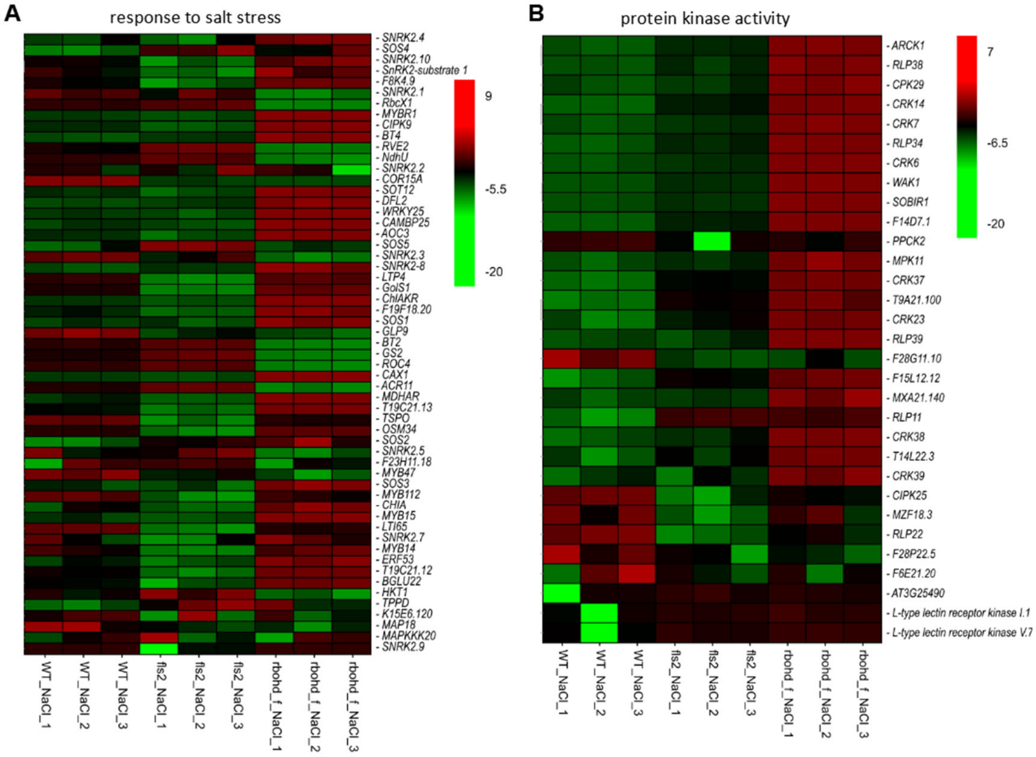

Figure S6. FLS2 and RBOHD are involved in regulating expression of genes related to salt stress and protein kinase under NaCl treatment. (A) and (B) Heatmap analysis of genes expression related to salt stress and protein kinase in WT, *fls2* mutant, and *rbohd rbohf* double mutant (rbohd_f) under NaCl treatment.

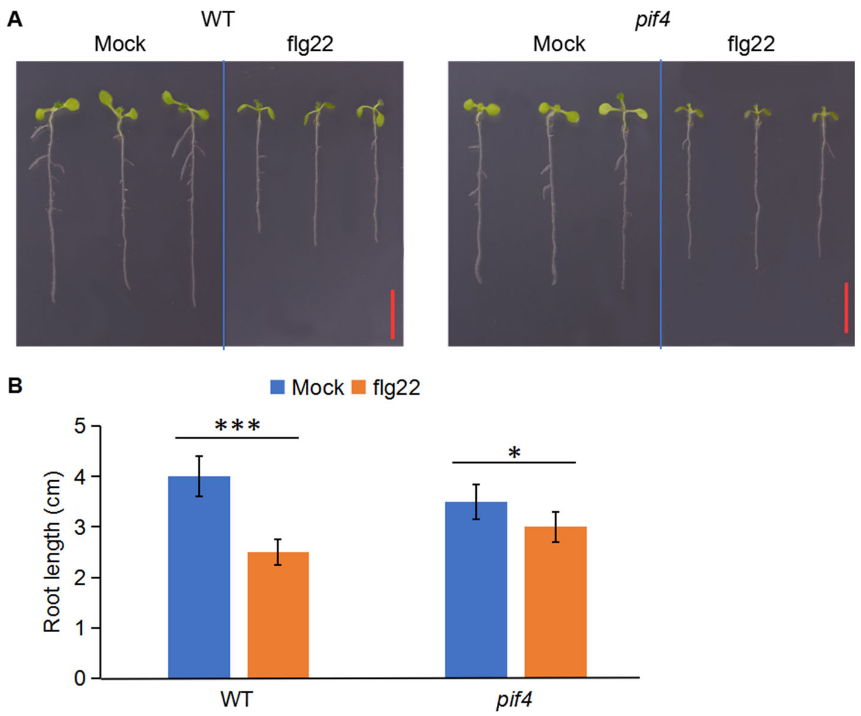

Figure S7. *pfi4* mutant shows decreased sensitivity to flg22. (A)Detection of the sensitivity of seedlings of WT and *pif4* mutant to flg22. (B) Statistical analysis of the root length of WT and *pif4* mutant seedlings after being treated with flg22, mock samples were used as controls. *: p<0.05, ***: p<0.001, student's t-test versus WT.
